# Editorial: Surfacing best practices for AI software development and integration in healthcare

**DOI:** 10.3389/fdgth.2023.1150875

**Published:** 2023-02-21

**Authors:** Mark Sendak, David Vidal, Sylvia Trujillo, Karandeep Singh, Xiaoxuan Liu, Suresh Balu

**Affiliations:** ^1^ Duke Institute for Health Innovation, Durham, NC, United States; ^2^ Mayo Clinic, Rochester, MN, United States; ^3^ OCHIN, Portland, OR, United States; ^4^ Division of Nephrology, Department of Internal Medicine, University of Michigan, Ann Arbor, MI, United States; ^5^ Academic Unit of Ophthalmology, Institute of Inflammation and Ageing, University of Birmingham, Birmingham, United Kingdom

**Keywords:** health innovation, health policy, technology regulation, technology diffusion, machine learning, artificial intelligence

**Editorial on the Research Topic** Surfacing best practices for AI software development and integration in healthcare

## Introduction

The evidence supporting the mainstream use of artificial intelligence (AI) software in healthcare is rapidly mounting. Three systematic reviews of AI software randomized controlled trials (RCTs) were published in 2021 and 2022, including 95 studies across 29 countries (1–3). In the United States (US), the Centers for Medicare and Medicaid Services (CMS) is approving AI software systems for reimbursement through multiple payment mechanisms (4). In the United Kingdom (UK), the National Screening Committee is exploring the use of AI software in national cancer screening and has awarded £90 million to prospective multi-center trials (5, 6). Two large, multi-hospital studies showed the mortality benefit of early detection and treatment of inpatient deterioration and pneumonia (7, 8). However, despite advances in technology and policy, isolated success stories are not leading to efficient diffusion of validated AI software across settings.

A key barrier preventing efficient translation of AI software to new clinical settings is the lack of visibility into poorly characterized, yet critically important labor that Mary Gray and Siddharth Suri call “Ghost Work” (9). Ghost work is broadly described as the invisible labor that powers technology platforms. In healthcare, ghost work is carried out by front-line clinical and administrative staff working beyond the contours of technical AI systems to effectively integrate the technologies into local social environments. But while the brittleness of AI software systems over time and across sites is broadly recognized (10, 11), health systems develop strategies largely in silos. To fill this gap, we invited teams from health systems around the globe to contribute to the research topic “Surfacing Best Practices for AI Software Development and Integration in Healthcare (12).” The research topic was sponsored by Janssen Pharmaceuticals of Johnson & Johnson. In this editorial, we present a synthesis of the nine featured manuscripts and highlight strategies used across settings as well as future opportunities for development and partnership.

## Methods

We conducted two primary analyses of the nine research topic manuscripts to identify key themes. We then complement the two primary analyses with details about the host institution, country, model use case, manuscript objectives, and key takeaways.

In the first primary analysis, we mapped the topics described in each manuscript to various stages of the AI software lifecycle. The four stages are defined as follows. First, *problem definition and solution procurement* describes the activities related to how organizations identify and prioritize problems and then allocate resources and personnel to pursue opportunities. Second, *AI solution development and adaptation* describes the activities related to how organizations either build technologies internally or adapt externally built tools. Third, *technical and clinical integration* describes the activities related to how organizations integrate AI solutions into legacy information technology systems and clinical workflows, roles, and responsibilities. Fourth, *lifecycle management* describes the activities related to maintenance, updating, and decommissioning of AI solutions used in clinical care. Each research topic manuscript could be mapped to multiple lifecycle stages.

In the second primary analysis, we reviewed biosketches, organization websites, and professional social media pages to map each research topic manuscript author to formal academic training across disciplines. Due to the large number of manuscript authors and broad range of formal training, we grouped disciplines into seven categories: engineering, computer science, and physics; statistics, biostatistics, and bioinformatics; business and management; public health and economics; biological or behavioral science; clinical doctorate; ethics or bioethics. Each author could be mapped to multiple academic disciplines.

## Results

The research topic “Surfacing Best Practices for AI Software Development and Integration in Healthcare” features 9 manuscripts with 73 authors from 7 institutions across 4 countries. Two institutions published two manuscripts each, including The Hospital for Sick Children in Toronto, Canada and University of Wisconsin in Madison, Wisconsin, USA. The AI software use cases featured in the research topic include three pediatric applications (hydronephrosis due to obstruction, arrhythmia detection, and sleep-wake patterns in neonates), one mental health application (suicide prevention), three general adult applications (30-day readmission, inpatient deterioration, and new-onset atrial fibrillation), and two geriatrics applications (advance care planning, falls risk in the emergency department). One research topic manuscript describes an organizational governance framework that has overseen ten AI software integrations, two decommissions, and one decision to not integrate (Liao et al.). Additional information about the use cases and key takeaways are presented in Table 1.

**Table 1:** Summary of key information from each research topic manuscript.

Name	Institution	Country	Model use case	Manuscript objective	AI translation phase	Key takeaways	Author domain expertise
An integration engineering framework for machine learning in healthcare	The Hospital for Sick Children	Canada	• “We have applied this framework to an arrhythmia detection model and have implemented this as a best practice at the Hospital for Sick Children. This practical application is demonstrated in the supplementary material.”	“Improper integration of new systems may lead to additional costs, patient harm, damage to other systems, and decrease in efficiency. To address this translation gap, we present a systems engineering framework to guide the development of models with explicit consideration of elements that are crucial for successful model integration in healthcare.”	Development; Technical Integration; Lifecycle management	• Applies systems engineering to the process of integrating machine learning in healthcare, describing the domains of integration (e.g., the technical system, human, and environment) and the interactions between the domains. • Conducted a narrative review to understand the challenges and gaps in integrating ML in health care, challenges associated with the current software development life cycle, and principles of integration engineering. • Present a generalizable framework for ML integration in health care with four phases: 1) inception, 2) preparation, 3) development, 4) integration.	Engineering, CS, or Physics: 8 Statistics, Biostatistics, or Bioinformatics: 0 Business or Management: 0 Public Health or Economics: 1 Biological or Behavioral sciences: 2 Clinical Doctorate: 6 Ethics or Bioethics: 1 **Total number of authors: 13** **Total number of domains: 5**
Clinical deployment environments: Five pillars of translational machine learning for health	University College London Hospital	UK	• “Our algorithm evaluates the risk of new onset atrial fibrillation (NOAF) in real-time based on existing electrolyte levels, medications, disease type, and the patient's co-morbidities… Our model is now used to drive a CDSS that operates in two layers. Firstly, where electrolytes are outside existing (evidence based guidelines) the CDSS makes a strong deterministic recommendation. Secondly, where electrolytes are within the window of the broader guideline but could be optimised, the CDSS makes a nudged randomised recommendation based on the model's prediction.”	“In this paper, we describe the functional requirements for a Clinical Deployment Environment (CDE) for translational ML4H. These requirements map closely to the classical components of translational medicine, but differ in that algorithms will require ongoing stewardship even after a successful deployment. The CDE is an infrastructure that manages algorithms with the same regard that is given to medicines (pharmacy) and machines (medical physics).”	Development; Technical Integration; Lifecycle management	• Presents five pillars to a clinical deployment environment for translating machine learning for health: 1) real world development; 2) ML-ops for health; 3) responsible AI in practice; 4) implementation science; 5) continuous evaluation. • Describes the similarities between translation of machine learning into healthcare and drug development. • Describes in great detail (in the supplement) the Experimental Medicine Application Platform (EMAP), where ML researchers iteratively build, validate, and test models, and the FlowEHR ML-Ops platform, which supports the deployment and maintenance of local models.	Engineering, CS, or Physics: 4 Statistics, Biostatistics, or Bioinformatics: 0 Business or Management: 0 Public Health or Economics: 1 Biological or Behavioral sciences: 0 Clinical Doctorate: 2 Ethics or Bioethics: 0 **Total number of authors: 6** **Total number of domains: 3**
The silent trial—the bridge between bench-to-bedside clinical AI applications	The Hospital for Sick Children	Canada	• “Classification model to predict obstruction in hydronephrotic kidneys of infants using ultrasound images” • “Develop an AI model that could reliably distinguish between self-resolving hydronephrosis vs. those that would ultimately require operative management based on initial kidney ultrasound”	“The purpose of this article is to highlight the lessons learned from our experience in validating a previously developed model within the context of the silent trial… Using our model as a case study, we illustrate how issues related to dataset drift, bias, feasibility, and stakeholder attitudes were identified and addressed. This article is intended for clinicians and ML engineers wishing to gain a deeper understanding of the rationale behind the silent trial and provide insights as to how this phase serves as a bridge between initial model development and clinical trials assessment.”	Technical Integration; Clinical Integration	• Describes a 2-step silent trial where first step is to assess generalization prospectively and second step retrained model and re-evaluated performance prospectively. • Describes approach to update model after silent trial to mitigate effect of dataset shift • Assessed patient and family perceptions about AI with post-visit follow-up questionnaire	Engineering, CS, or Physics: 3 Statistics, Biostatistics, or Bioinformatics: 2 Business or Management: 0 Public Health or Economics: 1 Biological or Behavioral sciences: 1 Clinical Doctorate: 3 Ethics or Bioethics: 1 **Total number of authors: 8** **Total number of domains: 6**
Operationalizing a real-time scoring model to predict fall risk among older adults in the emergency department	University of Wisconsin Health	USA	• “Our research team has developed and validated an innovative automated screening algorithm that uses machine learning coupled with electronic health record (EHR) data to predict fall risk in the 180 days following an ED visit using retrospective data (14).” • “This algorithm had the promise of identifying older adults at high risk of falling in the 6 months following the ED visit. Furthermore, engaging with experts in human factors engineering and clinicians, the study team designed a workflow and alerts designed to create a system in which the algorithm facilitates screening of older adult patients in the ED and facilitating referral for fall prevention services (15).”	“This case-study describes challenges and barriers we overcame in the use of such a model after it had been created and validated in silico. Based on this experience, we provide general principles for translating an EHR-based predictive model from research and reporting environments into real-time operation.”	Technical integration; Clinical Integration	• Detailed description of how the team made modifications to an algorithm through three stages over 15 months, including: stage 1) training and testing the algorithm on a research dataset; stage 2) validating the algorithm on production-system data; stage 3) live implementation of the algorithm. • Discussion of several unexpected technical challenges, including IT constraints to operationalize the model, model interpretability, model threshold selection, and model placement in the workflow.	Engineering, CS, or Physics: 2 Statistics, Biostatistics, or Bioinformatics: 0 Business or Management: 0 Public Health or Economics: 2 Biological or Behavioral sciences: 1 Clinical Doctorate: 1 Ethics or Bioethics: 0 **Total number of authors: 5** **Total number of domains: 4**
From compute to care: Lessons learned from deploying an early warning system into clinical practice	St Michael's Hospital	Canada	• “In Fall 2020, we deployed CHARTwatch to the General Internal Medicine (GIM) ward at St. Michael's Hospital, an inner-city teaching hospital in Canada.” • “We developed a model [CHARTwatch] to detect inpatient deterioration, defined as in-hospital death or transfer to the intensive care unit (ICU).”	“Here, we describe in detail, the system's infrastructure and assess the success of our deployment through quantitative metrics (such as model performance, end-user engagement, and adherence to workflows) and by comparing our deployment to the GMLP principles. The purpose of this manuscript is to provide concrete insights into the deployment of ML in a healthcare setting and highlight opportunities to strengthen GMLP guidance.”	Technical Integration; Clinical Integration	• Detailed description of the approach taken to minimize alert fatigue, including a 48 h snooze, 24 h snooze after ICU discharge, and silencing alerts after the fifth instance. • Description of changes made in response to a silent trial, including adapting model for high sensitivity troponin. • Presents best practices for downtime protocols (e.g., emails to IT team if script fails), end-user engagement, and training. • Presents concrete descriptions of how the team operationalized the 10 GMLP recommendations	Engineering, CS, or Physics: 1 Statistics, Biostatistics, or Bioinformatics: 1 Business or Management: 1 Public Health or Economics: 2 Biological or Behavioral sciences: 0 Clinical Doctorate: 2 Ethics or Bioethics: 0 **Total number of authors: 5** **Total number of domains: 5**
Considerations in the reliability and fairness audits of predictive models for advance care planning	Stanford Health Care	USA	• “In this work, we illustrate a reliability/fairness audit of 12-month mortality models considered for use in supporting team-based advance care planning (ACP) in three practice settings.”	“We (1) design and report a reliability/fairness audit of the models following existing reporting guidelines, (2) survey decision makers about how the results impacted their decision of whether to use the model, and (3) quantify the time, workflow and data requirements for performing this audit. We discuss key drivers and barriers to making these audits standard practice. We believe this may aid other decision makers and informaticists in operationalizing regular reliability and fairness audits.”	Lifecycle management	• Assessed two models, including an Epic end-of-life index and a homegrown 12-month mortality model, by performing a reliability audit (model performance and calibration) and fairness audit (summary statistics, subgroup performance, subgroup calibration). • Present audit results for both algorithms and determined differences in model performance and calibration across demographic subgroups. • Presents results of a survey sent to decision makers to understand the role of fairness and robustness audits in algorithmic governance. • Discuss the resource requirements and amount of effort (115 h) required to conduct the audit.	Engineering, CS, or Physics: 5 Statistics, Biostatistics, or Bioinformatics: 5 Business or Management: 1 Public Health or Economics: 3 Biological or Behavioral sciences: 2 Clinical Doctorate: 14 Ethics or Bioethics: 0 **Total number of authors: 27** **Total number of domains: 6**
Open questions and research gaps for monitoring and updating AI-enabled tools in clinical settings	Vanderbilt University Medical Center	USA	• “To explore performance drift in an operational setting, we evaluated the performance of two models currently implemented in the production EHR system at Vanderbilt University Medical Center (VUMC): a non-proprietary, externally developed model predicting readmission (LACE+) (23) and a locally developed model predicting suicidal behaviors (Vanderbilt Suicide Attempt and Ideation Likelihood model, VSAIL) (24).”	“In this paper, we highlight the need for maintaining clinical prediction models and discuss open questions regarding this critical aspect of the AI modeling lifecycle. First, we illustrate performance drift across models implemented in the production electronic health record (EHR) system at an academic medical center. Second, we discuss several open research questions and describe the nuances required for best practice guidance.”	Lifecycle management	• Presented two different types of model performance drift patterns using observed to expected outcome ratio (O:E) that occurred for a non-proprietary 30-day readmission model (LACE+) and for a homegrown suicide prevention model (VSAIL). • Detailed discussion of important questions related to three areas of lifecycle management: model maintenance policies (e.g., how should model ownership impact local control over maintenance?), performance monitoring perspectives (e.g., at what level should model performance be maintained? what aspects of performance should be monitored?), and model updating strategies (e.g., what updating approaches should be considered?).	Engineering, CS, or Physics: 0 Statistics, Biostatistics, or Bioinformatics: 3 Business or Management: 0 Public Health or Economics: 1 Biological or Behavioral sciences: 0 Clinical Doctorate: 2 Ethics or Bioethics: 0 **Total number of authors: 3** **Total number of domains: 3**
Governance of Clinical AI applications to facilitate safe and equitable deployment in a large health system: Key elements and early successes	University of Wisconsin Health	USA	• “At the time of this publication, the governance framework has overseen ten successful deployments, two successful retirements, and one successful non-deployment across nine applications.” • “Applications include diverse use of AI prediction for outputs including severe sepsis, clinical deterioration, physician panel weighting, COVID detection on radiographs, emergency department screening for falls prevention, screening for opioid abuse, and ED crowding”	“As we expand our technical ability to provide solutions, more skepticism and questions surface, and at times resistance, around the suitability of using AI in routine clinical care from all levels of the organization, ranging from front-line clinical staff to executive leadership. In response to these questions and the challenges for implementation, the health system recognized the need for a governance structure to endorse and oversee adoption, implementation, and ongoing value evaluation of AI-driven applications. This case study describes the development and nature of governance of clinical AI applications at our institution.”	Full lifecycle	• Describe clinical, operational, and leadership challenges encountered when establishing an institutional governance process. • Describes creation of institutional-level “Clinical AI and Predictive Analytics Committee” that oversees work conducted by use-case specific algorithm workgroups. • Presents five guiding principles that have emerged for the governance committee. • Describes approach for ongoing AI model monitoring, including a successful model retirement, and mechanisms to incorporate equity and ethics concerns related to AI	Engineering, CS, or Physics: 2 Statistics, Biostatistics, or Bioinformatics: 0 Business or Management: 0 Public Health or Economics: 3 Biological or Behavioral sciences: 0 Clinical Doctorate: 2 Ethics or Bioethics: 0 **Total number of authors: 4** **Total number of domains: 3**
A Perspective on a Quality Management System for AI/ML-Based Clinical Decision Support in Hospital Care	University Medical Center Utrecht	Netherlands	• “Sleep Well Baby…is an in-house developed ML model intended for monitoring real-time sleep-wake patterns in preterm neonates between 28- and 34-weeks gestational age.” • “The added value of real-time sleep-wake state monitoring comes from adapting elective clinical management of these preterm infants toward less disturbance during sleep periods.”	“In this perspective we illustrate our learnings regarding quality management of AI/ML-CDS tools through an example from our development pipeline, Sleep Well Baby (SWB). After introducing the SWB project and describing the development phase we address life-cycle management questions that arose while operationalizing SWB. When addressing these questions, we illustrate how the organizational structure of medical laboratories and ISO15189 can inspire healthcare institutes in building an effective and sustainable Quality Management System (QMS) for AI/ML usage in clinical care. Finally, in the discussion we provide an outlook how quality management of AI/ML-CDS extends to third-party AI/ML tools and settings outside healthcare institutes other than academic teaching hospitals.”	Full lifecycle	• Describes an innovation funnel process geared towards AI/ML product development process that became the blueprint for a national AI innovation tool. The funnel is divided into seven distinct phases with transition gates and references relevant EU-laws and regulations, guidelines, and standards. • Detailed description of how quality standards including IEC 62304 and ISO 14971 were applied during the development process of SWB. • Presents responses to highly relevant, practical life-cycle management questions: 1) Who is responsible for the AI/ML CDS device configuration; 2) Who gives clearance for the use of SWB in clinical practice?; 3) How to ensure safe change management and revision of SWB?; 4) What if model performance starts degrading?; 5) Who provides a helpdesk for users; 6) How are users trained?	Engineering, CS, or Physics: 1 Statistics, Biostatistics, or Bioinformatics: 1 Business or Management: 0 Public Health or Economics: 0 Biological or Behavioral sciences: 3 Clinical Doctorate: 3 Ethics or Bioethics: 0 **Total number of authors: 5** **Total number of domains: 4**

### AI software lifecycle stages

The research topic features manuscripts that contribute insights related to all four AI software lifecycle stages (problem definition and solution procurement, development and adaptation, technical and clinical integration, and lifecycle management). Two manuscripts describe programs that span all lifecycle stages, including the implementation of an AI quality management system at University Medical Center in Ultrecht, Netherlands and an AI organizational governance process at University of Wisconsin in Madison, USA. Two manuscripts present different frameworks for AI solution development, technical and clinical integration, and lifecycle management. A team from The Hospital for Sick Children in Toronto, Canada presents an approach that adopts language from systems engineering, while a team from University College London in the UK presents an approach that adopts language from therapeutics development (Assadi et al.). Two manuscripts present case studies focused on technical and clinical integration, including an adult deterioration model integrated at St. Michael's Hospital in Toronto, Canada, and a falls risk model integrated at University of Wisconsin in Madison, USA (Pou-Prom et al.). Lastly, three manuscripts present best practices related to specific lifecycle stages. A team from The Hospital for Sick Children in Toronto, Canada describes the use of AI software silent trials during technical integration (Kwong et al.), a team from Stanford Health Care in Palo Alto, USA describes reliability and fairness audits during lifecycle management (Lu et al.), and a team from Vanderbilt Health describes AI solution monitoring and updating during lifecycle management (Davis et al.).

### Team composition

In some ways, the research topic authorship teams are similar. All manuscripts feature interdisciplinary teams at academic health centers and graduate students and clinical trainees made significant contributions as co-authors. All manuscripts include clinical and technical expert co-authors. And lastly, all manuscripts build on prior work from authorship teams who have previously published AI solution validation studies.

In other ways, the research topic authorship teams are heterogeneous. The smallest teams were a group of three clinicians and informaticians at Vanderbilt Health who describe AI software monitoring and updating challenges and a group of four engineers, public health experts, and clinicians who describe the AI software organizational governance model at University of Wisconsin. The largest team was a group of twenty-seven engineers, bioinformaticians, managers, public health experts, biological science experts, and clinicians who conducted fairness and robustness audits of multiple models at Stanford Health Care. All teams included experts with formal training in at least three of the disciplines listed in Table 1 and two teams included experts with formal training in six disciplines. Among the 73 authors who contributed to the research topic, two perspectives were unique. There was a single AI ethics expert from The Hospital for Sick Children in Toronto, Canada and there was a senior data scientist at University Medical Center in Utrecht, Netherlands who is also a clinical microbiologist who has implemented and audited laboratory quality management systems.

## Discussion

The research topic “Surfacing Best Practices for AI Software Development and Integration in Healthcare” features a remarkably diverse set of insights and learnings from teams around the globe integrating and using AI software into practice (12). Throughout the research topic, teams consistently describe responses to unexpected challenges encountered in the transition from conducting AI software research to translating a technology into practice. The success of AI in healthcare hinges on the ability to adapt and transition from research into routine clinical practice. Sharing challenges, failures and describing promising approaches that were implemented in real-world settings can inform teams around the globe looking to advance the use of AI software in healthcare.

Across the research topic, consensus emerged around three important AI software integration practices. First, many teams highlighted the importance of simulating AI software performance in local, operational settings prior to initial use in clinical care. One method discussed in multiple articles involved the operationalization of a “silent trial,” during which bedside clinicians are initially blinded to the AI software as it is prospectively applied on operational data. While not novel, consensus is emerging around the importance of this activity (13–15). Silent trials can alert AI software developers to potential patient safety risks, bias, or integration concerns prior to clinical testing in a manner that minimizes risk to patients. Another article described the creation of a synthetic clinical deployment environment that anticipates real-world clinical decision making (Harris et al.).

Second, many teams highlighted the importance of AI software governance and management. Articles highlighted the importance of transdisciplinary teams and the need to assign responsibility and accountability to oversee AI software performance and appropriate use. One team used international standards to create a quality management system for AI software lifecycle management (Bartels et al.). Manuscripts in the research topic build upon existing frameworks and broaden the focus from AI software manufacturers to humans within health systems who oversee AI software used in clinical settings. The frameworks complement national efforts to equip the healthcare workforce to effectively adopt AI (16).

Lastly, many teams highlighted the importance of ongoing AI software monitoring and auditing. Some articles used existing standards for evaluating AI, including Health Canada/FDA/MHRA Joint Statement on 10 guiding principles for Good Machine Learning Practices (GMLP), however real-world experience led to additional recommendations, such as emphasizing user engagement, utilizing a silent trial, and creating downtime protocols. Another team described periodic reliability and fairness audits that went beyond quantitative comparison of AI software performance across demographic subgroups to also include stakeholder interviews to better understand the impact of the AI software.

While consensus emerged on the themes described above, the research topic did surface divergent perspectives on the importance of interpretability and explainability of AI software. For example, the teams at University of Wisconsin and University College London explicitly promote the use of explainable models. One team explained that “a desire to ensure we had an interpretable model further influenced our choice to pursue regression rather than tree-based models (Engstrom et al.).” The other team explained that “most AI models that operate as “black-box models” are unsuitable for mission-critical domains, such as healthcare, because they pose risk scenarios where problems that occur can remain masked and therefore undetectable and unfixable” (Harris et al.). This perspective offers a contrasting view from prior work examining the use of “black-box models” in clinical care (17), the limitations of current explainability methods (18), and the approach of regulators at the U.S. Food and Drug Administration (19). The research topic exposes the urgent need for research and policies that help organizations understand whether or not to prioritize AI software interpretability and explainability.

## Future directions

The research topic reveals five important opportunities to advance AI software integration in health care, summarized in Box 1. First, governments and health systems must invest in building and sustaining transdisciplinary teams that manage AI software integrations. Best practices did not emerge from the heroic acts of individual scientists, but rather from transdisciplinary teams of experts working with health systems. These types of roles are often funded through health system operations and require significant investment.

BOX 1 **Five recommendations that emerged from research topic manuscripts**

1) Governments and health systems must invest in transdisciplinary teams that manage AI software integrations
2) Health systems must broaden stakeholder engagement to include patients, legal and regulatory experts, and social scientists
3) Practitioner and research community must standardize AI software integration definitions, processes, and procedures, as well as communication approaches
4) Governments and health systems must establish durable, multi-stakeholder collaboratives to continue surfacing and disseminating AI software integration best practices
5) Governments must fund programs designed to foster the adoption of well-validated AI software beyond highly resourced academic health systems

Second, health systems must broaden stakeholder engagement throughout the AI software lifecycle. Unfortunately, only a single instance of direct patient engagement was described in the research topic, occurring at The Hospital for Sick Children in Toronto, Canada. Otherwise, there was limited patient and community engagement. And while the research topic authors were diverse, there was minimal representation of legal and regulatory experts and social scientists. These perspectives are crucial to ensure that AI software integration aligns with rapidly evolving regulations, and unintended consequences of AI software integration and use are anticipated, identified, and mitigated.

Third, there is an urgent need to develop and formalize standard AI software integration definitions, processes, and procedures as well as communication approaches (20). The research topic features teams that used language from different disciplines to describe AI software integration, including drug discovery, systems engineering, and international quality management standards. While it's important to build upon existing work across disciplines, the multiplicity of terms creates unnecessary ambiguity and confusion. Precisely defined steps and procedures need to be specified for rapid diffusion of more mature best practices, such as the “silent trial”.

Fourth, durable, multi-stakeholder collaboratives are needed to continue surfacing and disseminating AI software integration best practices. Efforts that we are directly involved in to achieve this aim are the Health AI Partnership (21) to disseminate best practices across health systems and the development of AI software reporting standards, including DECIDE-AI (22), CONSORT-AI (23), STARD-AI (24), and SPIRIT-AI (25).

Fifth, the research topic highlights the importance of fostering the adoption of well-validated AI software beyond highly resourced academic health systems. Persistence of the status quo, where AI software is best integrated within settings with the most expertise, will undermine the potential benefit of AI software. Business models and public sector programs must be designed to enable academic health systems to support smaller under-resourced settings that do not have the internal capabilities to utilize AI software most effectively. One research topic manuscript described a promising approach: “For smaller entities, such as a single general practitioner, this effort [to establish an AI software quality management system] seems unfeasible. In this situation, complete dependence on the manufacturer is imaginable, making it difficult to establish truly safe performance. Again, inspiration can be found in the regional services of medical laboratories that very often provide access to competences and resources for safe application of diagnostics. Regional AI labs could provide services for the development, acquisition, and quality control of AI/ML for smaller healthcare institutes including general practitioners (Bartels et al.).” Programs that test different approaches of regional, multi-institutional support are urgently needed to ensure equitable diffusion of AI software.

## Conclusion

The research topic “Surfacing Best Practices for AI Software Development and Integration in Healthcare” successfully surfaced best practices from 7 organizations across 4 countries. All teams were based at academic health systems and had previously published AI software validation studies. The research topic features insights across the AI software integration lifecycle and contributing authors represent diverse domains of expertise. There was consensus around the importance of local evaluations of AI software in a “silent trial”, establishing organizational governance structures for AI software, and monitoring of technologies post-integration. However, the research topic also exposed limitations of current work and we present five recommendations to further advance AI software integration across settings. We hope our work informs AI software developers and policy makers and contributes to future efforts to broadly engage stakeholders in multi-institutional learning collaboratives.

## References

[1] Zhou Q Chen ZH Cao YH Peng S. Clinical impact and quality of randomized controlled trials involving interventions evaluating artificial intelligence prediction tools: a systematic review. Npj Digit Med. (2021) 4(1):154. 10.1038/s41746-021-00524-2 34711955 PMC8553754

[2] Plana D Shung DL Grimshaw AA Saraf A Sung JJY Kann BH. Randomized clinical trials of machine learning interventions in health care. JAMA Netw Open. (2022) 5(9):e2233946. 10.1001/jamanetworkopen.2022.33946 36173632 PMC9523495

[3] Lam TYT Cheung MFK Munro YL Lim KM Shung D Sung JJY. Randomized controlled trials of artificial intelligence in clinical practice: systematic review. J Med Internet Res. (2022) 24(8):e37188. 10.2196/37188 35904087 PMC9459941

[4] Parikh RB Helmchen LA. Paying for artificial intelligence in medicine. Npj Digit Med. (2022) 5(1):63. 10.1038/s41746-022-00609-6 35595986 PMC9123184

[5] Taylor-Phillips S Seedat F Kijauskaite G Marshall J Halligan S Hyde C UK National screening committee’s approach to reviewing evidence on artificial intelligence in breast cancer screening. Lancet Digital Heal. (2022) 4(7):e558–65. 10.1016/S2589-7500(22)00088-7 35750402

[6] The Artificial Intelligence in Health and Care Award. Available at: https://transform.england.nhs.uk/ai-lab/ai-lab-programmes/ai-health-and-care-award/ (Accessed January 21, 2023).

[7] Dean NC Vines CG Carr JR Rubin JG Webb BJ Jacobs JR A pragmatic stepped-wedge, cluster-controlled trial of real-time pneumonia clinical decision support. Am J Resp Crit Care. (2022) 205(11):1330–6. 10.1164/rccm.202109-2092OC PMC9873107 35258444

[8] Escobar GJ Liu VX Schuler A Lawson B Greene JD Kipnis P. Automated identification of adults at risk for in-hospital clinical deterioration. N Engl J Med. (2020 Nov 12) 383(20):1951–60. 10.1056/NEJMsa2001090 33176085 PMC7787261

[9] Gray M Suri S. Ghost work. New York, United States: Harper Business (2019).

[10] Finlayson SG Subbaswamy A Singh K Bowers J Kupke A Zittrain J The clinician and dataset shift in artificial intelligence. New Engl J Med. (2021) 385(3):283–6. 10.1056/NEJMc2104626 34260843 PMC8665481

[11] Wong A Otles E Donnelly JP Krumm A McCullough J DeTroyer-Cooley O External validation of a widely implemented proprietary sepsis prediction model in hospitalized patients. JAMA Intern Med. (2021) 181(8):1065–70 . 10.1001/jamainternmed.2021.2626 PMC8218233 34152373

[12] Surfacing Best Practices for AI Software Development and Integration in Healthcare. Available at: https://www.frontiersin.org/research-topics/28021/surfacing-best-practices-for-ai-software-development-and-integration-in-healthcare (Accessed January 21, 2023). 10.3389/fdgth.2023.1150875 PMC9989472 36895323

[13] McCradden MD Anderson JA Stephenson EA Drysdale E Erdman L Goldenberg A A research ethics framework for the clinical translation of healthcare machine learning. Am J Bioeth. (2022) 22(5):8–22. 10.1080/15265161.2021.2013977 35048782

[14] Wiens J Saria S Sendak MP Ghassemi M Liu VX Doshi-Velez F Do no harm: a roadmap for responsible machine learning for health care. Nat Med. (2019) 25:1337–40. 10.1038/s41591-019-0548-6 31427808

[15] Sendak MP Ratliff W Sarro D Alderton E Futoma J Gao M Real-World integration of a sepsis deep learning technology into routine clinical care: implementation study. JMIR Med Inform. (2020) 8(7):e15182. 10.2196/15182 32673244 PMC7391165

[16] Horizon Scanning. Available at: https://digital-transformation.hee.nhs.uk/building-a-digital-workforce/dart-ed/horizon-scanning (Accessed January 21, 2023).

[17] Sendak MP Elish MC Gao M Futoma J Ratliff W Nichols M The human body is a black box”: supporting clinical decision-making with deep learning. FAT* ‘20: conference on fairness, accountability, and transparency; vol. 44 (2020). p. 99–109. Available from: https://dl.acm.org/doi/pdf/10.1145/3351095.3372827?download=true

[18] Ghassemi M Oakden-Rayner L Beam AL. The false hope of current approaches to explainable artificial intelligence in health care. Lancet Digit Health. (2021) 3(11):e745–50. 10.1016/S2589-7500(21)00208-9 34711379

[19] Ross C. A “disaster”, or a “clear path” forward?: New FDA guidance on AI in medicine sparks strong reactions. STAT News. (2022). Available from: https://www.statnews.com/2022/09/28/fda-artificial-intelligence-tools-regulation-oversight/ (Accessed January 21, 2023).

[20] Sendak MP Gao M Brajer N Balu S. Presenting machine learning model information to clinical end users with model facts labels. npj Digit Med. (2020) 3(41):1–4. 10.1038/s41746-020-0253-3. 32219182 PMC7090057

[21] Duke Institute for Health Innovation. Health AI Partnership: an innovation and learning network for health AI software. (2021). Available from: https://dihi.org/health-ai-partnership-an-innovation-and-learning-network-to-facilitate-the-safe-effective-and-responsible-diffusion-of-health-ai-software-applied-to-health-care-delivery-settings/ (Accessed January 21, 2023).

[22] Vasey B Clifton DA Collins GS Denniston AK Faes L Geerts BF DECIDE-AI: new reporting guidelines to bridge the development-to-implementation gap in clinical artificial intelligence. Nat Med. (2021) 27(2):186–7. 10.1038/s41591-021-01229-5 33526932

[23] Liu X Cruz Rivera S Moher D Calvert MJ Denniston AK Keane P. Reporting guidelines for clinical trial reports for interventions involving artificial intelligence: the CONSORT-AI extension. Nat Med. (2020) 26:1364–74. 10.1038/s41591-020-1034-x 32908283 PMC7598943

[24] Sounderajah V Ashrafian H Aggarwal R Fauw JD Denniston AK Greaves F Developing specific reporting guidelines for diagnostic accuracy studies assessing AI interventions: the STARD-AI steering group. Nat Med. (2020) 26(6):807–8. 10.1038/s41591-020-0941-1 32514173

[25] Rivera SC Liu X Chan AW Denniston AK Calvert MJ Ashrafian H Guidelines for clinical trial protocols for interventions involving artificial intelligence: the SPIRIT-AI extension. Br Med J. (2020) 370:m3210. 10.1136/bmj.m3210 32907797 PMC7490785

